# Multimodal Learning for Mapping the Genotype-Phenotype Dynamics

**DOI:** 10.21203/rs.3.rs-4355413/v1

**Published:** 2024-05-16

**Authors:** Farhan Khodaee, Rohola Zandie, Elazer R. Edelman

**Affiliations:** 1 Institute for Medical Engineering and Science, Massachusetts Institute of Technology, Cambridge, 02139, MA, USA.; 2 Department of Medicine (Cardiovascular Medicine), Brigham and Women’s Hospital, Boston, 02115, MA, USA.

**Keywords:** integrated genetics, multimodal foundation model, language modeling, single-cell RNA sequencing, polyfunctional gene, transformer

## Abstract

How complex phenotypes emerge from intricate gene expression patterns is a fundamental question in biology. Quantitative characterization of this relationship, however, is challenging due to the vast combinatorial possibilities and dynamic interplay between genotype and phenotype landscapes. Integrating high-content genotyping approaches such as single-cell RNA sequencing and advanced learning methods such as language models offers an opportunity for dissecting this complex relationship. Here, we present a computational integrated genetics framework designed to analyze and interpret the high-dimensional landscape of genotypes and their associated phenotypes simultaneously. We applied this approach to develop a multimodal foundation model to explore the genotype-phenotype relationship manifold for human transcriptomics at the cellular level. Analyzing this joint manifold showed a refined resolution of cellular heterogeneity, enhanced precision in phenotype annotating, and uncovered potential cross-tissue biomarkers that are undetectable through conventional gene expression analysis alone. Moreover, our results revealed that the gene networks are characterized by scale-free patterns and show context-dependent gene-gene interactions, both of which result in significant variations in the topology of the gene network, particularly evident during aging. Finally, utilizing contextualized embeddings, we investigated gene polyfunctionality which illustrates the multifaceted roles that genes play in different biological processes, and demonstrated that for VWF gene in endothelial cells. Overall, this study advances our understanding of the dynamic interplay between gene expression and phenotypic manifestation and demonstrates the potential of integrated genetics in uncovering new dimensions of cellular function and complexity.

## 1 Introduction

Understanding the intertwined relationship of genotype and phenotype has long captivated biologists. Wilhelm Johannsen was the first person who coined the terms and attempted to quantify them. In his book “Elemente der exakten Erblichkeitslehre” (1909) [johannsen1909elemente], he attempted to provide an “exakten” description to biological phenomena by relating genotype to phenotype, both conceptually and mathematically. A century later, we still remain significantly distant from achieving a precise quantification of this relationship. In recent years, however, new sequencing techniques, such as single-cell RNA-seq (scRNA-seq), have propelled our understanding forward. These technologies reveal the complex dynamics of gene expression at a cellular resolution, highlighting the vast landscape of genotypes influenced by myriad factors [norman2019exploring]. However, these techniques fail to provide a comprehensive mapping of how genotypic combinations give rise to emerged phenotypes.

Current methods in mapping the genotype-phenotype relationship such as forward or reverse genetics, while theoretically capable of dissecting this relationship, fall short in practice due to the sheer scale and complexity involved [replogle2022mapping]. In human cells, a combination of thousands of genes gives rise to an incredibly diverse phenome landscape [freimer2003human]. Furthermore, the advent of scRNA-seq, while groundbreaking, introduces its own set of challenges. The technique’s ability to uncover thousands of gene expression changes across cells offers a detailed view of the transcriptomic landscape but also complicates the task of drawing meaningful biological conclusions from these high-dimensional datasets [kiselev2019challenges, stegle2015computational]. This complexity is exacerbated by the vast amounts of data generated in the field, necessitating advanced computational techniques for analysis.

Recent developments in machine learning, particularly the adaptation of self-supervised transformer architectures from the field of natural language processing (NLP), have shown promise in analyzing complex biological datasets [theodoris2023transfer, cui2024scgpt]. In these approaches, each gene is a word and each cell state can be modeled as a sentence equivalent to natural language. Current models, however, often lack the interpretability needed to fully understand the sources of variability and fail to capture the dynamic interplay between genotypes and related phenotypes. They primarily focus on resolving cellular heterogeneity without addressing the multifaceted nature of gene expression patterns, which are not only cell-type specific but also highly responsive to organism-level phenotypic characteristics such as age and sex.

Here, we propose the application of self-supervised language models to map the genotype-phenotype landscape simultaneously and introduce the concept of integrated genetics as opposed to forward and revere genetics. In this approach, the phenotypes such as sex, age, anatomical tissue and cell types are integrated and learned with genotypic information measured by scRNA-seq data. This enhances our understanding of the biological context of gene expression and ultimately the genotype-phenotype relationship. In particular, we apply this approach to Tabula Sapiens cell atlas [the2022tabula], creating an integrated foundation model, called PolyGene, comprised of nearly 500,000 human cells across 24 organs from various age ranges. Our results show that PolyGene successfully learns genotype-phenotype annotations and also offers new insights into contextualized gene network dynamics. Ultimately, our work lays the groundwork for a phenotype-informed deep learning framework that can be fine-tuned for a broad range of downstream applications, potentially revolutionizing our understanding of the genotype-phenotype relationship and accelerating the discovery of therapeutic targets.

## 2 Results

### 2.1 Model Overview

PolyGene is a multimodal foundational model to extend the application of attention-based deep learning methods in scRNA-seq data analysis. Our model leverages the unique characteristic of language models, which is their ability to integrate multimodal information by incorporating phenotypic metadata. Multimodal machine learning offers several unique advantages [jayagopal2022multimodal], such as enhanced learning for a more comprehensive understanding of the data [kline2022multimodal], improved robustness in dealing with noisy or incomplete data [cheng2023robustness], and cross-modal understanding between different data modalities. These advantages enable the model to perform tasks that require bridging the gap between various types of data [lin2023multimodality]. Therefore, we hypothesize that our method can lead to a more accurate biological contextualization, where a better understanding of variation sources and heterogeneity is necessary.

**Fig. 1:**
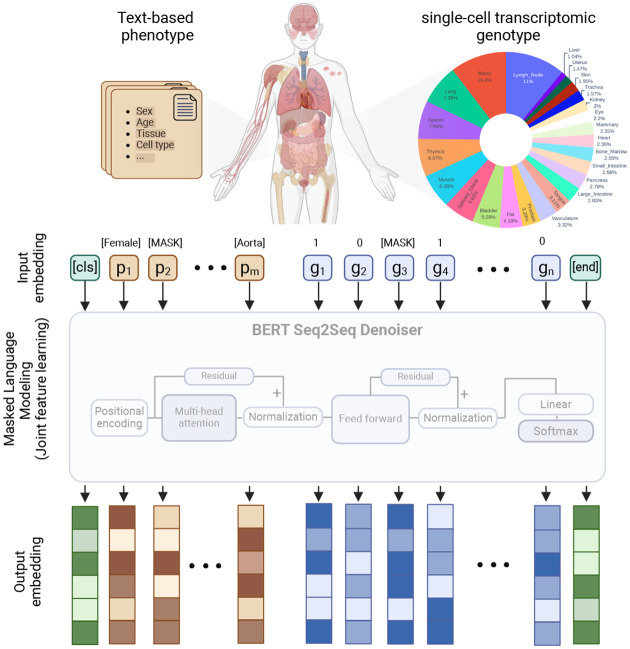
Overview of the model. Gene expression data from single-cell RNA-seq and associated phenotypes are embedded as input to a language model. The model learns the genotype-phenotype relationship in an integrated manner, where genes and phenotype are masked simultaneously in the input layer. The outputs of the model are high-dimensional embeddings which are information-rich

In our approach, for each cell, the gene expression values and associated phenotypes are embedded into a vector representation and passed into the network (4). We have incorporated different data modalities of the information of single-cell transcriptomes as well as corresponding phenotypes where each gene expression was measured and encoded jointly as the inputs to the model (figure1). With this structure, PolyGene can learn the relationship between phenotypes (sex, age, tissue, and cell type) and contextualized phenotype-genotype relationship. Furthermore, PolyGene develops a more meaningful biological understanding of the expression levels in the context of its preceding neighbors’ rankings, and of the phenotype in which that gene has appeared. We refer to this method as integrated genetics, where forward genetics and reverse genetics are accomplished concurrently by masking genotype and phenotype during learning. The resulting outputs of the model, integrated embeddings, include rich information about the contexts in which the genes or phenotypes have appeared.

### 2.2 Resolving heterogeneity in phenotypes: Cell type, tissue, and age annotation

**Fig. 2:**
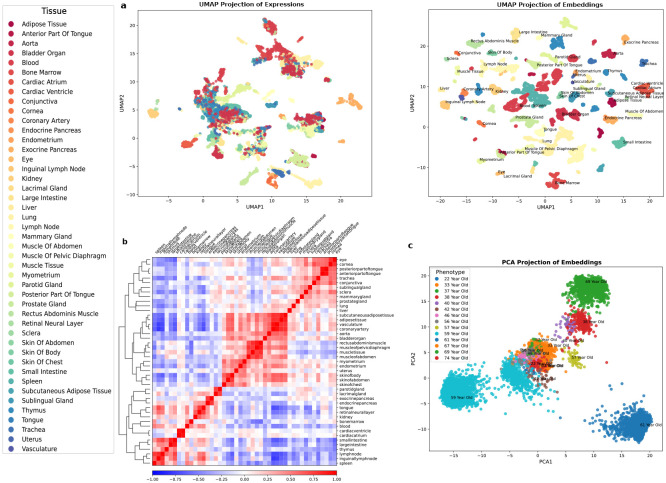
Resolving heterogeneity in phenotypes using contextualized embeddings. **a**, UMAP dimension reduction visualization of human cell atlas, colored by tissue type, using gene expression data (left), using output embeddings on the same sample cluster tissue more distinctly (right). **b**, Pearson correlation between tissues’ embeddings demonstrates a hierarchical grouping of different tissues, similar tissues have a higher correlation. **c**, linear dimension reduction on age embeddings separates different age groups clearly

One of the main roadblocks in interpreting scRNA-seq expression data is the presence of a high degree of heterogeneity. Cellular heterogeneity stems from the inherent diversity of individual cells within a population or variability in the experimental setup and sequencing techniques. Understanding the source of this heterogeneity is crucial for extracting meaningful insight, from basic cellular processes to complex physiological functions. To address this, we have integrated metadata describing the context of gene expressions with the data. This metadata encompasses a wide array of variables, including the specific cell type, organ of origin, and donor demographics such as age and sex. By incorporating these descriptors, our model enhances the precision of cell-specific embeddings, thus facilitating a more nuanced understanding of cellular heterogeneity. This refinement in the model’s capability to accurately represent individual cells has led to a substantial improvement in clustering performance (figure 2a). Dimension reduction on contextualized embeddings significantly improves the characterization of cell types, the tissue of origin, and even the age and sex of the donors with notable distinctions (figure 2c and figure S3). Further, the classification of these contextualized embeddings has unveiled the presence of two distinct subclusters within several tissues, including the thymus, trachea, subcutaneous adipose tissue, and the parotid gland (figure 2b). This finding demonstrates the model’s sensitivity to subtle intratissue variations, potentially indicative of distinct functional states or developmental stages. Moreover, our analysis highlights a pronounced heterogeneity within the blood and lung tissues, which suggests a higher degree of cellular diversity in these tissues. This observation aligns with the complex functional roles and dynamic environments associated with these tissues.

Next, we explored the potential of contextualized embeddings in analyzing tissue similarities. By leveraging these high-dimensional embeddings, we conducted a comprehensive examination of the relationships between different tissue types using Pearson correlation coefficients. The results demonstrate a landscape of tissue similarity with encoded biological relationships (figure 2c). An example is the high similarity score observed between the cardiac ventricle and cardiac atrium tissues which aligned with their close functional and anatomical relationships. The tissue similarity results also reveal an unexpectedly high similarity between the tongue, the retinal neural layer, and the kidney. This finding, although initially unexpected, aligns with previous research indicating the tongue’s diagnostic relevance for chronic kidney disease [chen2021tongue, bemelman2014failing]. This intricate link highlights the potential of cross-tissue biomarkers in systemic disease diagnosis. The ability of multimodal integrated genetics to independently discover such relationships indicates its utility in revealing novel biological insights beyond traditional analysis methods.

Beyond individual pairwise comparisons, our analysis has revealed a structured hierarchy within the tissue similarity landscape. Specifically, muscle tissues emerge as a distinct cluster, demonstrating inherent similarities among themselves. Yet, this cluster is not isolated; it forms part of a larger conglomerate that includes the aorta, vasculature, and uterus (figure 2b). This broader aggregation suggests a deeper, developmentally rooted connection between these tissues, hinting at shared origins or functional pathways. We observed a categorization of all examined tissues into three major subgroups. Except for some abnormalities, these subgroups align well with the primary germ layers from which they originate i.e. mesoderm, endoderm, and ectoderm. These results demonstrate the model’s potential in understanding tissue differentiation and organogenesis based on embedding vectors.

### 2.3 Embeddings encode a dynamic genotype-phenotype relationship

Integrated genetics methodology creates contextualized embedding outputs which can serve as multidimensional representations of genotypic and phenotypic data, encapsulating the intricate associations and variations within biological contexts. The high-dimensional characteristics of the embeddings allow novel analysis of specific phenotypic traits or states associated with certain genotypic profiles. To understand what genes universally have influenced the phenotypes, we calculated the cosine similarity between phenotype embeddings and gene embeddings (Extended data 4). Our results showed that H4C3 (H4 clustered histone 3) is the most prominent gene among all the phenotypes. For tissue phenotype, urokinase plasminogen activator receptor (PLAUR) and transcription factor XBP1 are among the most similar genes observed in all tissues (Extended data 4a). XBP1 is also observed in cell type, age and with lesser impact in sex phenotype. For cell types, however, lysosome C (LYZ) gene has the highest universal similarity (Extended data 4b). Follistatin (FST) gene is one of the most similar genes to the age phenotype embedding which shows the role of tissue activin and cellular proliferation in aging (Extended data 4c).

Further analysis identified differentially embedded genes (DEGs) among phenotypes. Our results showed that for tissue phenotype, IGHV3–7, CCL17 and IGKV6D-21 are the DEGs (Extended data 4e). For cell type phenotype, CCL3 (C-C Motif Chemokine Ligand 3, CA1 (Carbonic anhydrase 1) and CCL4L2 (C-C motif chemokine 4-like) are the top different genes between all cell types (Extended data 4f). The differentiating genes in age phenotype are RGS1 (regulator of G-protein signaling 1), CA1 and CCL3 (Extended data 4g), and for sex phenotype CA1, CCL17 (CC chemokine ligand 17) and EREG (Epiregulin) are shown to be embedded significantly different between male and female (Extended data 4h). Overall, the DEGs show that immune-related genes play the most important role in differentiating the embeddings of various phenotypes. In particular, CA1 and CCL17 molecules play a larger role in differentiating contextualized phenotypes.

We hypothesized that embeddings represent an information-rich representation of each phenotype and genotype. Focusing on tissue phenotype, the embedding analysis distinctly separated tissue subtypes (3a). Specifically, for different muscle cells, genes like DNAJB1, GEM, and GPRC5A were identified as similar across different muscle tissues (3b), while HMGN2P40, CCNB1, and CTD and GNLY genes were notably different (3c). This methodology extended to other tissues, with vasculature and cardiac tissues examined for the impact of genotypes on emergent phenotypes (3). For instance, for cardiac tissue, the maternally expressed gene 3 (MEG3) gene was identified as the top DEG that distinguishes ventricle embeddings from atrium embeddings. Previously, this gene has been implicated in ventricular dysfunctions such as myocardial fibrosis and compensatory cardiac hypertrophy, conditions that predominantly affect ventricular rather than atrial tissue. [piccoli2017inhibition, zhang2019stat3, mi2023inhibition]. Notably, kidney and tongue tissues were found to share significant similarities, attributed to genes such as transcription factor ELF3 (which regulates epithelial cell differentiation), CRYAB (molecular chaperone in skeletal muscle), LGALS4, PDK4 (highly expressed in skeletal muscle), and CD59 (expressed in smooth muscle cells), highlighting the molecular variations among difference muscle cells. Overall, our results demonstrate how high-dimensional embedding analysis can help with understanding the genetic diversity contributing to phenotypic heterogeneity.

### 2.4 Contextualized genotype encodings across phenotypes

Next, we leveraged gene embeddings to characterize how specific genes exhibit varied functions and associations across different phenotypes, specifically organs. Our investigation into IL2 gene embeddings unveils a unique pattern of tissue similarity. Notably, clustering based on IL2 embeddings shows the similarity between the inguinal lymph node, bone marrow, and the skin of the chest and abdomen, setting them apart from other lymph nodes and skin regions across the body (4). This distinction suggests potential multifaceted roles of IL2 across different tissues, and its involvement in diverse biological processes, specifically to induce similar immune responses in T and B lymphocytes at these tissues [boyman2012role]. Additionally, IL2 embeddings delineate a clear difference between the large and small intestines. Previous studies in mice have demonstrated that IL-2 is necessary for maintaining immunologic homeostasis in the large intestine, but is not essential in the small intestine, indicating the existence of different sources of IL-2 [zhou2019innate]. This provides a compelling example of how a single gene’s influence can diverge significantly across closely related tissues.

On the contrary, the analysis of KRT8 (Keratin 8) gene embeddings presents a different scenario. Here, the small and large intestines exhibit a high degree of similarity when clustered based on KRT8 embeddings, aligning more closely with traditional understandings of tissue similarity (4. This outcome points out the heterogeneous nature of gene function across tissues, with KRT8 demonstrating a more uniform pattern of influence within the intestinal tract. We have explored this polyfunctional characteristic of genes in the next section in more detail.

To demonstrate that the genotypes are dynamic and context-dependent, we examined the distance between gene embeddings in various tissues and cell types. This examination aimed to uncover how the same genes manifest different degrees of similarity or divergence within and across biological contexts. For instance, for aorta, spleen, and subcutaneous adipose, certain genes—NICOA7, XBP1, BNIP3L, and CTSD—exhibited distinct patterns of similarity. These genes, despite being expressed across the examined tissues, showed closer embedding proximity between the aorta and subcutaneous adipose than between the aorta and spleen. This suggests a more nuanced interaction of these genes within the cardiovascular and fat storage systems compared to their roles in the immune function represented by the spleen. Conversely, the embedding distances for TIMP1, CD55, and HSPA5 across these tissues were notably less variable, indicating a more uniform functional role or expression pattern across diverse biological systems.

Expanding our analysis to cell types further revealed the dynamic nature of gene embeddings. The FBXO7 gene, known for its multifunctional role across various physiological processes, showed significantly closer embeddings between memory B cells and naive B cells than between memory B cells and kidney epithelial cells. This observation highlights the similarity in FBXO7’s function or interaction network within B cells, in contrast to its role in epithelial cells. On the other hand, the SMS gene, which encodes the spermine synthase enzyme, maintained a consistent embedding similarity across B cells and epithelial cells, suggesting a universal role that transcends cell type specificity.

### 2.5 Gene Network Structure is Context-dependent

A significant contribution of our work is the illumination of dynamic gene networks that operate across different phenotypic contexts. The integration of phenotypes allowed for identifying embedded gene expression patterns and context-dependent interactions that can be also investigated in the structure of the gene interaction networks. By including cell metadata in our model, we can encode the conditions in which gene expression occurs and sources of variation into the gene embeddings. In this section, we characterize the relationship between these gene embeddings in different contexts.

First, we will analyze the overall structure of gene networks across different phenotypes. Metabolic, protein, and gene interaction networks have been reported to exhibit scale-free behavior which makes them robust and efficient in processing information [khanin2006scale, arita2005scale]. However, the context-dependent architecture of the network from RNA expression data has not been reported before. Here we used similarity between gene embeddings to construct the gene network (where nodes are genes edges are the similarity score higher than a specific threshold). Our results show that the networks follow a scale-free pattern, where the distribution of connections to nodes follows a power law (Extended data 5). This indicates that most nodes have few connections, while a small portion of important nodes have a huge number of connections (hubs). The degree distribution results across various cell types demonstrate the scale-free nature of all networks, albeit with the phenotype-dependent variation that manifests itself in the slope of power law (Extended data 5). Additionally, the construction of gene interaction dendrograms facilitates a deeper understanding of the networks’ hierarchical organization. These dendrograms reveal clusters of genes with similar embeddings, each representing a distinct functional unit, such as signaling pathways or cellular response mechanisms (Extended data 5).

Specifically, we investigated the role of aging in the structure of genetic network in endothelial cells (ECs). Our results show that aging alters the slope of the power-law distribution within the genetic network, with a pronounced effect on low-degree nodes—those with fewer connections (5a). These findings reveal a significant restructuring of the network. Specifically, we observed that specialized nodes, which typically have fewer connections, begin to form more linkages, thereby diluting their specialized roles within the network (5b) To pinpoint the genes most susceptible to these age-related structural changes, we delved into differentially embedded genes within aged ECs. Our analysis highlighted several genes such as KCNH8 (potassium voltage-gated channel subfamily, Kv12.1), DNAJA4 (DnaJ Heat Shock Protein Family (Hsp40) Member A4), and EGLN3 (Egl-9 Family Hypoxia-Inducible Factor 3) as exhibiting the most significant alterations in their network embeddings. These genes are proposed as candidates for further investigation into their roles in vascular aging, potentially offering new insights into the molecular underpinnings of age-related vascular changes.

### 2.6 Polyfunctional gene embeddings represent genetic heterogeneity

The extraction of high-dimensional gene embeddings from our model offers a unique perspective into the diverse contexts and functions where genes are active. Through these embeddings, we can gain an insight into the functional heterogeneity of genes, which we call polyfunctional genes. This is defined as the gene’s ability to form multiple, functionally distinct clusters even within the same cell type. Contextualized embeddings reveal the complex regulatory landscapes that genes navigate, the distinct networks that they construct, and potentially the multifaceted roles they play in various biological processes.

To demonstrate the concept of the polyfunctional gene, we have analyzed VWF (von Willebrand Factor) gene within endothelial cells. We identified two functionally distinct clusters that are not phenotype dependent (Extended data 6): the first aligns with VWF’s well-documented role in blood coagulation and critical function in hemostasis (cluster 1 in 6a). In contrast, the second cluster reveals a lesser-known aspect of VWF’s functionality, related to its involvement in the management of reactive oxygen species and the cellular response to glucocorticoid stress (cluster 0 in 6a)). The mechanism of this polyfunctional manifestation could be RNA splicing, which has been observed previously to play an important role in mediating the expression of VWF in endothelial cells [yuan2013role, hawke2016characterization].

Similarly, our examination of the CD55 gene, known for its regulatory role in the complement system, across T cells presents a similar pattern of functional diversity. The embeddings reveal six distinct clusters (6b), each representing different aspects of CD55’s function or suggesting variations in the gene’s expression sources within the same cell type. These clusters range from their canonical role in protecting cells from complement-mediated lysis to potentially novel functions that warrant more investigations. This diversity within the gene embeddings in a specific cell type highlights the existence of complex, multifunctional roles that a single gene can embody, which challenges our conventional understanding of gene function. However, polyfunctionality is not a universal characteristic of all genes. For instance, we showed that for transcription factor KLF5, no distinct functional clusters were observed in endothelial cells (Extended data 7). In general, genes that encode surface proteins show higher diversity in their embeddings compared to genes that translate to intracellular proteins.

These findings illustrate the power of contextual gene embeddings in uncovering the broad spectrum of functional heterogeneity inherent in genes. By dissecting these polyfunctional clusters, our method enriches the gene function lexicon and opens new avenues for exploring the dynamic interplay between genes and their expression contexts. This research on the complex roles of genes enhances our understanding of biology and sets the stage for future studies on the molecular mechanisms that influence cellular behavior and organism physiology. One of the main applications of polyfunctionality could be in improving the precision of targeting the special function of a gene in drug discovery and cell therapy.

## 3 Discussion

A central goal of genetics is to understand the complex relationship between gene expression profiles and emergent phenotypes. However, studying this relationship is challenging because of the vast array of possibilities and the bidirectional nature of the relationship, which necessitates a holistic approach. Here, we introduce the concept of integrated genetics to navigate the genotype-phenotype landscape simultaneously and provide a multimodal foundational model to accomplish it for human transcriptomics. By modeling these landscapes jointly, we can gain insights into the coordinated mechanisms underlying genetic expression and phenotypic manifestation.

**Fig. 3:**
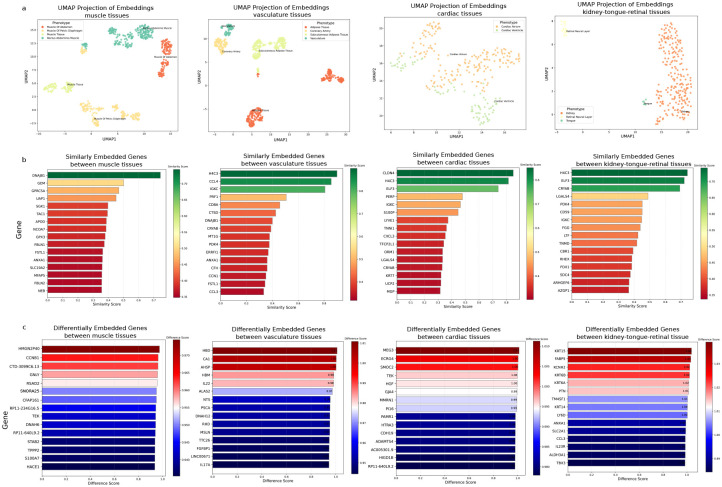
Contextualized embeddings from integrated genetics help to reveal intricate genotype-phenotype relationship. **a**, UMAP visualization of tissue phenotype embeddings for various tissue types that are considered similar such as muscle, vasculature, cardiac, and tissues are considered different such as kidney, tongue, and retinal tissue. **b**, similarity analysis between phenotype embeddings of tissue and genotype embeddings present in those tissues can identify genes that have similar roles in these tissues. **c**, similarity analysis between various tissues can help with identifying differentially embedded genes that have been different in all observed contexts

**Fig. 4:**
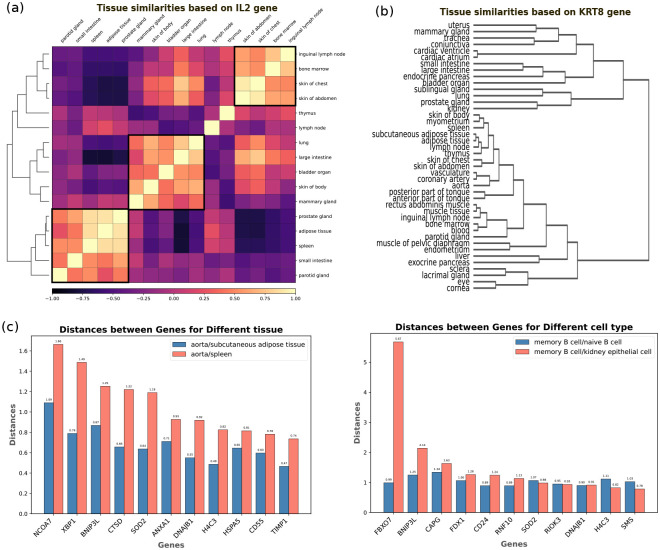
Dynamic phenotype relationships. Measuring similarity for gene embeddings in different contexts reveals a dynamic relationship between phenotypes. **a**, analysis of IL2 embeddings demonstrates a pattern of similarity between tissues that do not specifically correlate with the anatomical location of the tissues. **b**, KRT8 embeddings show a different pattern from IL2 which is more similar to the anatomical organization of the tissues. **c**, evaluating Euclidean distance between gene embeddings for different tissues (left) and cell types (right) demonstrates that gene embeddings are context dependant

**Fig. 5:**
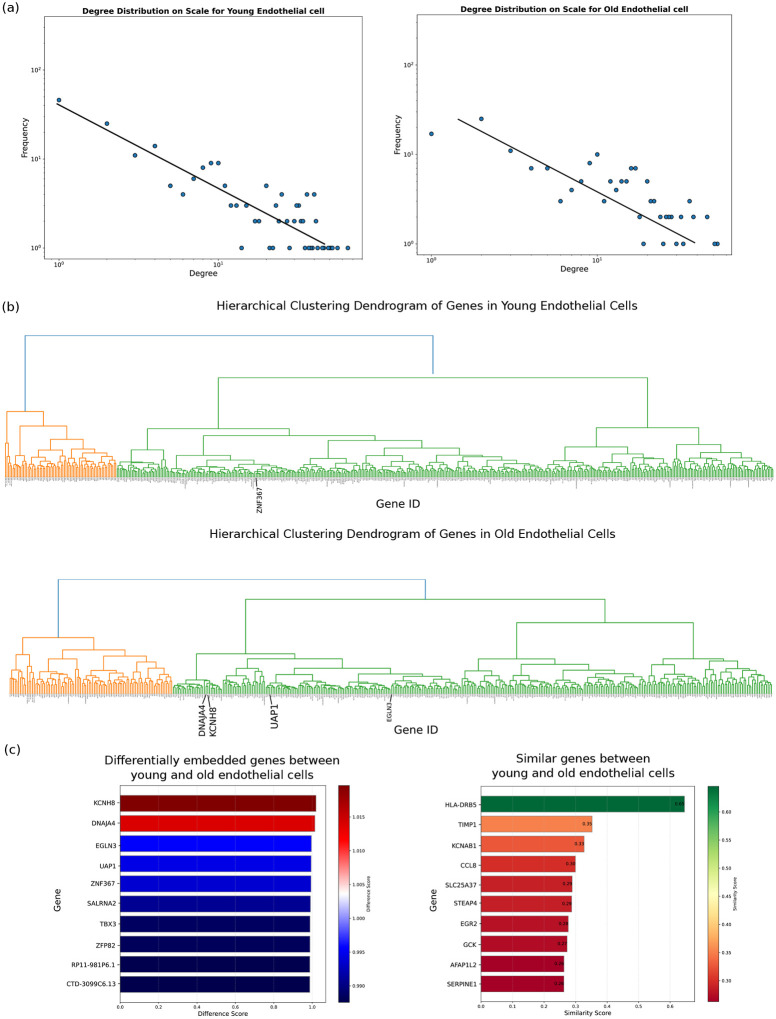
Aging restructures the gene network in endothelial cells. **a**, degree distribution analysis on endothelial cells shows a scale-free pattern with different characteristics of the networks for endothelial cells of donors under 40 years old (left), and cells from donors more than 60 years old (right). **b**, Clustering of gene embeddings demonstrates a hierarchical structure in the network highlighting the changes in subnetworks between young endothelial cells (top) and old endothelial cells (bottom) which is more pronounced in the specialized region of the network (depicted by orange color). Genes that are highly different between the two networks are scaled for visibility. **c**, similarity analysis between gene embeddings reveals differentially embedded genes between young and old endothelial cells (left) and genes with a higher degree of similarity that do not change during vascular aging (right)

**Fig. 6:**
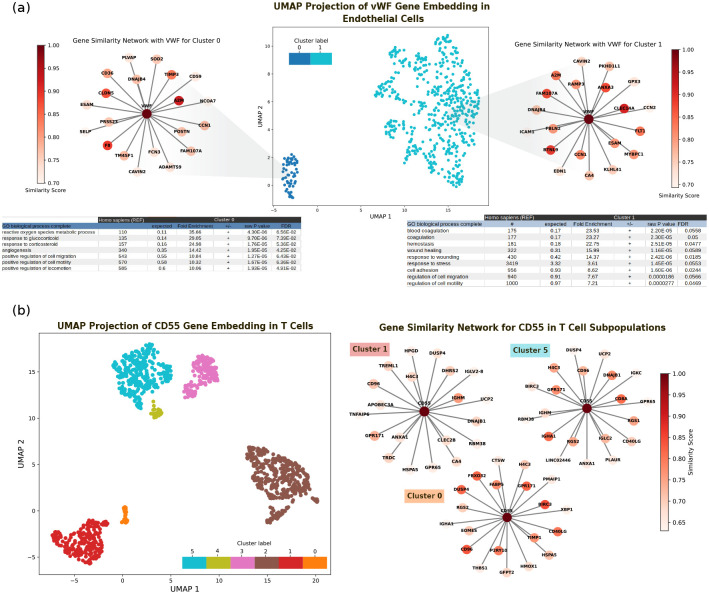
Contextual gene embeddings capture polyfunctional characteristics in VWF in endothelial cells and CD55 in T cells. **a**, Clustering of VWF gene’s embeddings in endothelial cells reveals two distinct clusters (middle). In cluster 0, VWF interacts with genes that are involved mostly in reactive oxygen species metabolic process (lef), and cluster 1 shows the more canonical function of VWF in blood coagulation. **b**, Contextualized embeddings for CD55 gene in T cells show 6 distinct clusters (left), the networks that clusters form for clusters 0, 1, and 5 are depicted (right)

We demonstrated that the resultant embeddings from our approach contain meaningful information about the genotype-phenotype relationship. Specifically, analyzing embeddings can resolve cellular heterogeneity and significantly refine the accuracy of phenotype annotations, facilitating advanced clustering and similarity analysis demonstrated in fig 1. At the tissue level, our analysis shows potential cross-tissue biomarkers and subtle intra-tissue variations that can be used for systemic disease diagnosis (illustrated in fig 2 and fig 3). Our study highlights the phenotype-dependent nature of gene networks (fig 4), revealing scale-free patterns of the gene network (shown in fig Extended data 5). Specifically, we demonstrated the phenotype-dependant variations in the gene network structure for endothelial cells during aging and proposed potential genes involved (fig 5). Additionally, we explored the polyfunctionality of genes through contextualized embeddings and demonstrated the diverse roles a single gene can play in various biological processes (demonstrated for VWF and CD55 genes in fig 6). The tissue-specific polyfunctional characteristics of the genes provide greater insight into the side effects observed when targeting genes clinically; thus, our analysis could aid in designing more accurate and safe therapeutics.

Our study aims to decode the intricate genotype-phenotype relationship by integrating content-rich transcriptomics data and advanced machine learning. By incorporating various factors such as cell type, tissue of origin, and donor demographics into our analysis, we have substantially improved the precision of pattern recognition. This integration allows for the use of information-rich, high-dimensional embeddings that offer new analytical capabilities and enhance our understanding of genetic dynamics. The insights derived from our multimodal foundational model open up possibilities for precision medicine to exploit individual genetic profiles, enabling the development of customized therapeutic strategies. Such strategies could be utilized to improve efficacy and minimize adverse effects by predicting potential cross-tissue side effects. Furthermore, this approach holds the promise of integrating more clinical data modalities both related to observed phenotypes and measured genotypes. This could significantly alter therapeutic development, leading to more effective, safer, and finely targeted interventions that are optimized for individual genetic and phenotypic profiles, thereby dramatically improving patient outcomes.

## 4 Methods

In this section, we will provide an overview of the PolyGene architecture and key features. Previous works [yang2022scbert, theodoris2023transfer, cui2024scgpt] on gene expression data from scRNA-seq data have been limited to statistical analysis of a large number of genes, for example, clustering and cell type annotation. This approach mirrors the pre-2010 natural language processing (NLP) research, which primarily focused on clustering, classification, topic modeling, and other statistical analyses. However, the advent of word embedding catalyzed a shift towards more granular text analyses, such as Named Entity Recognition (NER), sentiment analysis, and semantic interpretation [mikolov2013distributed]. We advocate for a parallel methodological evolution in genomics, leveraging the exponential growth in genetic data availability across various species. Concurrently, the integration of advanced machine learning frameworks, notably the Transformer architecture’s success in NLP and related domains, has paved the way for analogous applications in genetics.

It’s been known for a long time that there is a connection between genes in cells in terms of a network also known as Gene Regulatory Network (GRN). Despite this, research in this domain has predominantly been theoretical, with rudimentary models like Stuart A. Kauffman’s Random Boolean Networks (RBN) focusing on the overarching characteristics of these networks under varying conditions, such as network stability contingent on nodes and connectivity [kauffman2003random, shmulevich2004activities]. In essence, the methodology to elucidate the complex interrelationships among genes that result in intriguing phenotypic manifestations—phenomena that single gene analyses cannot account for—remained elusive.

The limitations of existing models employing Transformer [vaswani2017attention] architectures in genomics arise from their reliance on a constrained subset of genes, organized by their expression levels from highest to lowest. This ordering premise is problematic for several reasons. Firstly, it presumes, without clear justification, that a descending expression-based sequence represents a gene’s natural or functionally relevant order. More critically, this method’s validity is compromised by its sensitivity to expression value disparities; minor fluctuations or noise can alter the gene sequence, thereby impacting the analysis. This issue is not encountered in text analysis, where the inherent sequential nature of language enables Transformer models to extract meaningful patterns by evaluating the distance between tokens. For instance, in the textual context, the change of word order in phrases like “it is clear” to “is it clear” significantly shifts the meaning. This natural order is not inherently present in gene expression data.

A potential solution for the positional embedding problem in genetics is its complete elimination, yet this introduces new challenges in loss function computation. The challenge stems from the ambiguity in comparing input tokens with output when the input is permutation invariant. One approach to address this is by generating numerous permutations of the input to refine loss calculation or by adopting methodologies like Set-Transformer. However, these solutions are inefficient for practical implementation due to their computational demands. In our study, we adopt an innovative strategy, employing a consistent positional encoding for each gene, complemented by padding for unexpressed genes within a specific cell. This method effectively resolves the issues associated with loss function computation while circumventing the constraints imposed by gene order.

### 4.1 Input representation

To model our problem we start with a representation of the genes in a single cell *C* that includes all of them as a vector. For the sake of consistency and without loss of generality, we select a specific and fixed order for these genes:

(1) $$C=\left[g_{1},g_{2},\ldots ,g_{N}\right]$$

Each gene value is the expression of the gene in that cell that is normalized to a range. To make the values of the expression comparable, we have to normalize the value by dividing it by the dispersion rate. To choose a threshold for distinguishing expressed from non-expressed genes, we conduct a histogram analysis of gene expression values across all cells. This analysis reveals a significant concentration of expression values below 0.1, followed by an increase, forming a distinct bump in the histogram. Such a distribution pattern indicates that a threshold of 0.1 is an effective demarcation for identifying genes as expressed or non-expressed.

After determining the threshold, the rest of the expression range is divided into *B* equal-sized bins and each gene is represented as the number of the corresponding bin (higher expression corresponds to higher bins). More specifically each gene value belongs to a bin:

(2) $$g_{i}\in [0,B-1]$$

Given the extensive number of genes, the resultant input vector length, if every gene is included, would be impractically large for efficient computation. To address this, our initial step involves sorting the genes based on their “variability” or the degree to which their expression levels differ across various cells. We then assign a rank to each gene according to this variability criterion. This ranking process enables us to prioritize genes based on their variability, thereby streamlining the input size by focusing on those genes that are likely to provide the most informative insights into cellular functions and states. We can model each cell by *T* highly variable genes (from all *N* genes):

(3) $$C=\left[g_{1},g_{2},\ldots ,g_{T}\right]\;T\leq N$$

To pass the genes into the network we need to first embed them in a vector representation. A gene embedding is constructed by mapping its value which is the bin number into an initially random embedding of size *h*. This way, the cell is represented as a matrix **X** as follows:

(4) $$X_{h\times T}=\left[X_{1},X_{2},\ldots ,X_{N}\right]$$

In which **X**_*i*_ is of size *h* × 1 and is defined as:

(5) $$X_{i}=\text{embedding}\left(g_{i}\right)$$

### 4.2 Model architecture

To calculate the contextualized embedding of each gene, a denoiser also known as BERT (Bidirectional Encoder Representations from Transformers) [devlin2018bert] has been employed. The model is dependent on both directions of the input (unlike autoregressive models that are only dependent on the previous words/tokens). In this study, a cell, denoted as **X** and characterized by a sequence of genes, undergoes a process to generate its corrupted counterpart, **X**˜. This corruption involves the random masking of the proportion *r* of its genes. This procedure can be equivalently conceptualized as independently and randomly masking each gene within the sequence.


(6) $$\tilde{X}=\text{Bernoulli}(r)\;r\in [0,1]$$

The task of *MLM* (Masked Language Modeling) is to reconstruct the expression values of masked genes based on the known genes. The corrupted version is then fed into the Transformer model.

The Transformer models is decomposed of *L* layers of Transformer blocks *H*_*θ*_:

(7) $$X^{l}=H_{\theta }\left(X^{l-1}\right)\;l\in [1,L]$$

The Transformer block takes an input of length *T*, and outputs the same size in the output:

(8) $$H_{\theta }(X)=\left[H_{\theta }\left(X_{1}\right),H_{\theta }\left(X_{2}\right),\cdots ,H_{\theta }\left(X_{T}\right)\right]$$

In which *H*_*θ*_ is the composition of the following functions:

(9) $$\bar{X^{l}}=\text{LayerNorm}\left(X^{l}\right)\;M^{l}=\text{MultiHead}\left(\bar{X^{l}}\right)+X^{l}\;\bar{M^{l}}=\text{LayerNorm}\left(M^{l}\right)\;X^{l+1}=\text{FeedForward}\left(\bar{M^{l}}\right)+M^{l}$$

LayerNorm and FeedForward are the usual layers used in modern neural networks. MultiHead function is the multi-head attention module that is defined as follows

(10) $$\text{MultiHead}(X)=\text{Concat}\left(z_{1},\ldots ,z_{k}\right)W_{o}$$

(11) $$z_{i}=\text{Attention}\left(XW_{i}^{Q},XW_{i}^{K},XW_{i}^{V}\right)$$

(12) $$\text{Attention}(Q,K,V)=D^{-1}AV,\;A=exp\left(QK^{T}/\sqrt{d}\right),\;D=\text{diag}\left(A1_{L}\right)$$

where *Concat*(.) is the concatenation function, and the *diag*(.) is a diagonal matrix with the input vector as the diagonal. Also, **1**_*L*_ is the all-ones vector of length *L* and *exp*(.) is applied element-wise. All the matrices $W_{i}^{Q}$, $W_{i}^{K}$, $W_{i}^{V}$ and **W**_0_ are trainable parameters.

It should be noted that **W**_*p*_ is the position embedding that in the first layer of the Transformer maps the discrete inputs into initial random embeddings, in order to distinguish each gene we rely on its position. Note that, unlike text, there is no natural order in gene sequences. So here the position embedding makes it easy to distinguish between each gene. **W**_*e*_ is the gene embedding for each expression. The size of position embedding is *B*. The input to the first transformer block is:

(13) $$X_{0}=GW_{e}+W_{p}$$

In which *G* is the binned matrix. The last layer of the Transformer is passed through a feedforward layer and results in the “logits” that will be passed through a softmax function to get the probabilities over the expression values.


(14) $$S_{\theta }\left(\hat{X}|{\tilde{X}}^{1}\right)=X^{L}W_{e}^{T}\;p_{\theta }\left(\hat{X}|{\tilde{X}}^{1}\right)=\frac{\text{exp}\left(S_{\theta }\left(\hat{X}|{\tilde{X}}^{1}\right)\right)}{\sum_{\hat{Z}}\text{exp}\left(S_{\theta }\left(\hat{Z}|{\tilde{X}}^{1}\right)\right)}$$

The MLM task is to minimize the cross entropy between the probability distribution from the model and the hard labels (one hot encoding) from the dataset:

(15) $$\underset{\theta }{\text{min}\;}\text{CrossEntropy}\left(q,p_{\theta }\right)=-\overset{B}{\underset{i=1}{\sum }}q\;\text{log}\;p_{\theta }$$

Because one hot encoding has been used the equation 15 will reduce to:

(16) $$\underset{\theta }{\text{max}}\;\text{log}\;p_{\theta }(\hat{X}|\tilde{X})\approx \overset{T}{\underset{t=1}{\sum }}I_{M}(t)\;\text{log}\;p_{\theta }\left({\hat{X}}_{t}|\tilde{X}\right)$$

In which *I*_*M*_(*t*) is the indicator function for the masked tokens (*M*). Here, the independence assumption between the prediction of each gene has been made.

This methodology is pivotal as it enables the extraction of contextualized embedding for each gene, considering the interactions and relations with other genes within the same cell. By capturing the nuanced interplay among genes, this approach allows us to construct a more refined and dynamic model of the gene interaction network. Additionally, a significant advantage of this method over preceding approaches is that the computation of the loss function is not contingent on the precision of gene ranking.

When the number of bins is larger than 2 (*B* > 2), we are dealing with Ordinal regression. The original implementation of the BERT doesn’t have this problem because there is no natural order in the tokens but expressions are binned into an ordered list. For this case, we have to change the loss function to take into account this subtlety. Our approach to solving this problem is using the soft labeling technique. In this technique instead of focusing all the probability on the label (one hot encoding above) we take *r* percent of it and distribute it equally to other bins, so each other bin has $\frac{r}{B-1}$ probability. Even though this doesn’t account for the distance (the bin number 9 is closer to 8 than bin number 1) it still helps the network to converge faster.

To address this challenge, we employ a soft labeling technique. Traditionally, a one-hot encoding scheme would allocate a probability of one to the true label and zero to all others. Soft labeling, however, moderates this approach by redistributing a portion of the probability across all bins. Specifically, we allocate a fraction *r* of the probability to the true label, while the remaining probability is evenly dispersed among the other bins, with each receiving a probability of $\frac{r}{B-1}$. This method doesn’t explicitly account for the ordinal proximity between bins (e.g., bin 9 is closer to bin 8 than to bin 1); nevertheless, in practice it has the same effect because it facilitates faster network convergence by providing a gradient across adjacent categories, thereby enhancing the learning process regarding the ordinal relationship between bins.

### 4.3 Multi-Task Learning: Integrating Genotype and Phenotype

One of the biggest challenges faced by traditional approaches of modeling genomics data is the problem of relating the Genotype and Phenotype. These relationships are not often very straightforward, linear, or intuitive to understand. In this paper, we use Multi-task learning to understand this problem better. Multi-task learning is an approach in machine learning that focuses on improving generalization in transfer learning using different signals in the training data. Some of the seemingly unrelated signals in the data can be used as inductive biases to learn a shared representation that helps wildly different tasks.

Each cell has other phenotypic features such as sex, tissue type, cell type, developmental stage, and diseases. The formula 1 is expanded and the input to the model can be represented as follows:

(17) $$C=\left(p_{1},p_{2},\ldots ,p_{F},g_{1},g_{2},\ldots ,g_{N}\right)$$

in which *p*_*i*_’s are the phenotypes and *C* represents a cell as the input to the model. The model is designed to perform a multi-task learning approach, where the objective is to not only predict the gene expressions but also the phenotypic values at the same time. The masking probability for phenotypes and genotypes are different: phenotypes are masked with higher probability because the number of phenotypes is lower than the genotypes and this helps the model to be more robust. In our experiments, we used %50 for phenotypes masking probability versus %15 for genotypes.

This approach has been very successful in other domains of machine learning such as computer vision and NLP [raffel2020exploring], but their power has not been used extensively in the domain of genomics data. There are several advantages to this technique, for example, it helps to generalize better in phenotypic domains where there is data scarcity. Also, it gives much more flexibility in exploring a joint model that incorporates both phenotypic and genotypic features where we can control and observe the effects of each phenotype on gene expressions and vice versa.

### 4.4 Pre-training

In this study, we implemented an unsupervised learning strategy by employing a masking algorithm. The input data is randomly masked and a model is trained to make predictions based on the remaining inputs. We randomly mask the non-zero gene expression and then reconstruct the original inputs by model predictions using the remaining genes. This approach essentially enables the model to learn predictive patterns and relationships within the data, fostering a deeper understanding of the gene interactions and dependencies by attempting to reconstruct the original input based on the partial information available.

### 4.5 Evaluation and Predictions

Three different model sizes are trained based on the top highly variable genes. The largest model (PolyGene 2432) encompasses all the highly variable genes identified in the original dataset. The other two models (PolyGene 512 and 256) incorporate top 512 and 256 highly variable genes respectively. We evaluated the precision (P), recall (R), and F1 for each model across all the phenotypes, and all the phenotypes and genotypes (all the input data). Notably, the main difference in performance can be seen in phenotypes which can be attributed to reduced predictive ambiguity in the phenotypic domain when compared to gene expression analysis, as evidenced in the confusion matrix.

### 4.6 Implementation Details

The models were optimized by the Adam optimizer, using a mini-batch size of 10, at a starting learning rate of 2e-5, with a warmup ratio of 0.1. They are trained for a total of 13 epochs. We used the Scanpy [wolf2018scanpy] python library for gene expression pre-processing, including normalization, log-transformation and highly variable gene selection. We also used pytorch and huggingface [wolf2019huggingface] library for model design and training.

## Extended Data

**Fig. Extended data 1:**
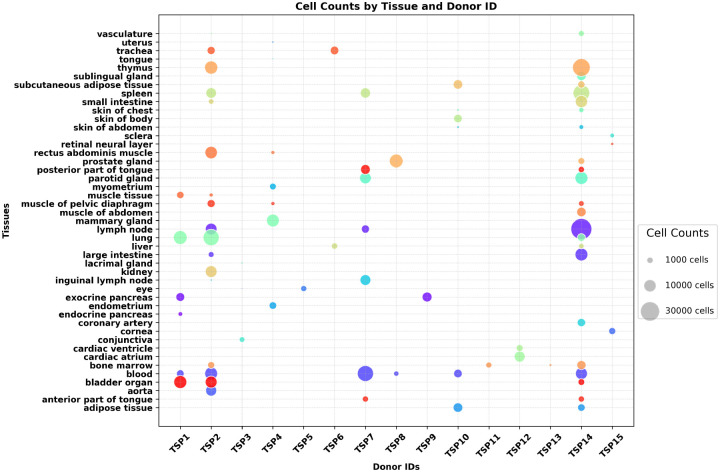
Overview of Tabula Sapiens dataset. The human atlas includes nearly 500,000 cells from 24 different tissues listed. More than 400 cell types from 15 donors with different demographics are included in a multimodal dataset. For more details about the Tabula dataset refer to [the2022tabula]

**Fig. Extended data 2:**
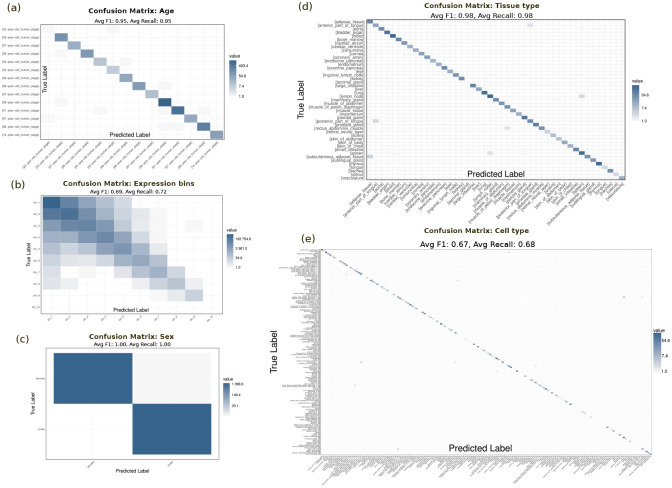
Predicted vs true labels matrix for various phenotypes and gene expression binning. **a**, confusion matrix for age phenotype. **b**, confusion matrix for expression bins. **c**, confusion matrix for sex phenotype. **d**, confusion matrix for tissue type phenotype. **e**, confusion matrix for cell type phenotype.

**Fig. Extended data 3:**
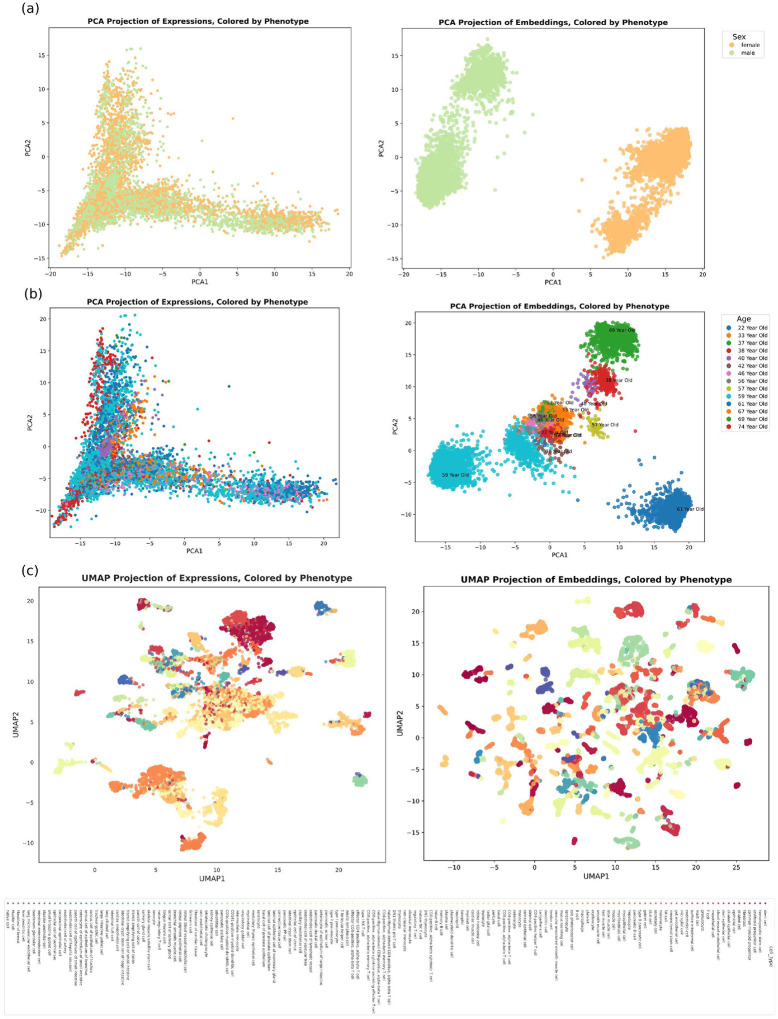
Visualization of embeddings compared to expressions The embeddings obtained from the integrated foundation model show a higher resolution of cellular heterogeneity. **a** Principle Component Analysis (PCA) on gene expressions (left) and embeddings (right) colored by sex phenotype. **b** PCA on gene expressions (left) and embeddings (right) colored by age phenotype. **a** UMAP visualization of gene expressions (left) and embeddings (right) colored by cell type phenotype.

**Fig. Extended data 4:**
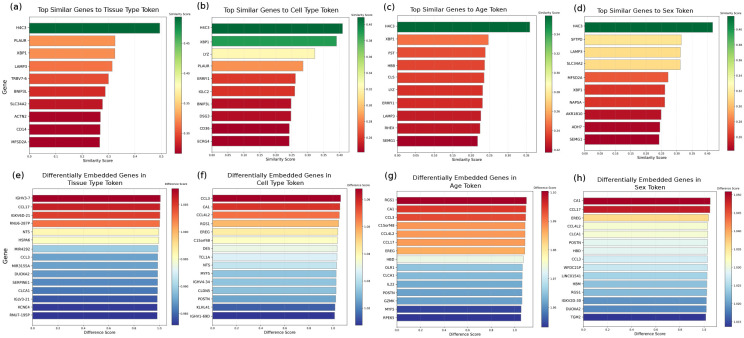
Similarity analysis on phenotype embeddings **a-d** Top similar genes to tissue, cell, age and sex phenotypes. **e-h** Top different genes to tissue, cell, age and sex phenotypes

**Fig. Extended data 5:**
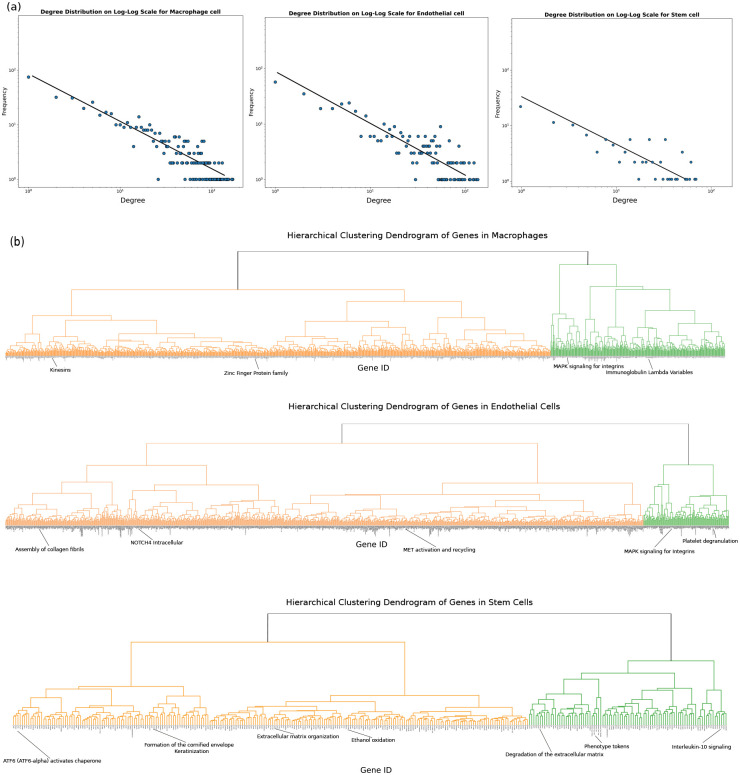
Gene network constructed from the gene embeddings shows scale-free and hierarchical structure. **a**, power-law degree distribution shown for different cell types: macrophages (left), endothelial cells (middle), and stem cells (right). **b**, Clustering of gene embeddings shows a hierarchical structure of gene interaction network which differs between various cell types: macrophages (top), endothelial cells (middle), and stem cells (bottom)

**Fig. Extended data 6:**
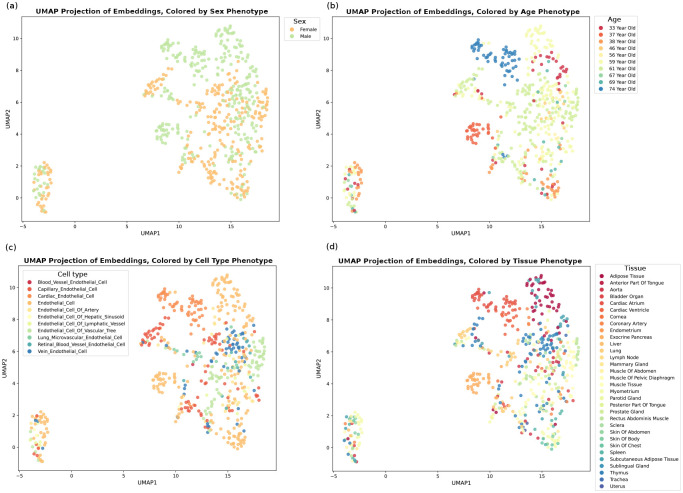
VWF gene in endothelial cells shows polyfunctional characteristics which are not phenotype-dependant. **a**, UMAP visualization of VWF embedding shows no difference between males and females. **b**, polyfunctionality is conserved during aging. **c**, endothelial cells (capillary, vein, etc) from different tissues show VWF polyfunctionality. **d**, VWF’s embedding shows a tissue-independent polyfunctionality

**Fig. Extended data 7:**
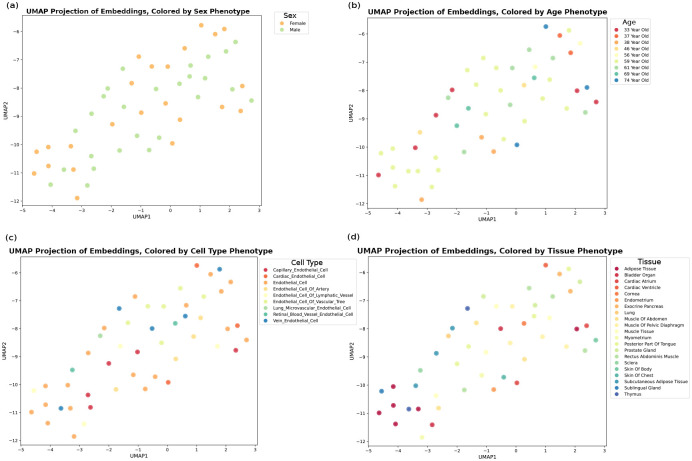
KLF5 gene in endothelial cells does not show polyfunctional characteristics. **a-d** UMAP visualization of the KLF5’s embeddings show not distinct clusters in different sexes, ages, cell types or tissue types

## Figures and Tables

**Table 1:** Comparing the performance of different model sizes of PolyGene for phenotypes and also phenotypes and genotypes

Model	P (Phenotype)	R(Phenotype)	F1 (Phenotype)	P (all)	R (all)	F1 (all)
PolyGene 2432	86.96	86.18	86.56	76.22	73.48	74.82
PolyGene 512	85.23	84.17	84.69	76.47	72.32	73.38
PolyGene 256	79.13	75.66	77.35	75.57	70.27	72.82

## Data Availability

We incorporated scRNA-seq data from the Tabula Sapiens study [the2022tabula] which provides manually annotated cell and tissue type, and also includes metadata about each cell. The data includes nearly 500,000 cells from 24 different tissues and organs, many from the same donor. Annotation of cell types shows more than 400 cell types of the human body in 14 different donors. The dataset includes transcriptomics from the bladder, blood, bone marrow, eye, fat, heart, kidney, large intestine, liver, lung, lymph node, mammary, muscle, pancreas, prostate, salivary gland, skin, small intestine, spleen, thymus, tongue, trachea, uterus, and vasculature.
